# The Work of the Elizabeth Barclay Home of Industry

**Published:** 1917-03-03

**Authors:** 


### The Work of the Elizabeth Barclay Home of Industry.

Although four years have passed since the Mental Deficiency Act was placed on the Statute-book, the Elizabeth Barclay Home of Industry at Bodmin is still the only institution for the care of the feeble-minded in the whole county of Cornwall. Probably other institutions would have been established had not the war intervened, but for the present the Cornish county authority, like others, feels unable to face the expense of opening a certified home for its defectives. The Board of Control, therefore, recognising the useful part the Elizabeth Barclay Home is playing, has granted a certificate of approval for five years, and Sir Frederick Needham, the senior commissioner, expresses thankfulness that such a home exists in Cornwall. The institution meets—unfortunately only for a very limited class—the case of girls and women who stand in need of training in useful occupations, so as to preclude them becoming a burden and anxiety to their families. During the past year the girls have been much interested in helping to prepare sphagnum moss and in making comforts for soldiers. The report of the institution, presented at the annual meeting recently by Miss Shaw, the secretary, mentioned that in a great majority of the 1,100 cases inquired into by the County Mental Deficiency Committee the great care and devotion shown by the relations to feeble-minded persons are very striking, but the outstanding need is training centres and voluntary homes where a useful occupation can be taught.

